# Clinical, Operational, and Economic Benefits of a Digitally Enabled Wound Care Program in Home Health: Quasi-Experimental, Pre-Post Comparative Study

**DOI:** 10.2196/71535

**Published:** 2025-04-08

**Authors:** Heba Tallah Mohammed, Kathleen Corcoran, Kyle Lavergne, Angela Graham, Daniel Gill, Kwame Jones, Shivika Singal, Malini Krishnamoorthy, Amy Cassata, David Mannion, Robert D J Fraser

**Affiliations:** 1 Swift Medical Inc Toronto, ON Canada; 2 CenterWell Home Health Greenwood, IL United States; 3 Summit Health Boston, MA United States; 4 Arthur Labatt Family School of Nursing Western University London, ON Canada

**Keywords:** home health care, artificial intelligence, AI, digital wound care, wound assessment, operational efficiency, clinical outcomes, healing time, cost saving, skilled nursing visits

## Abstract

**Background:**

The demand for home health care and nursing visits has steadily increased, requiring significant allocation of resources for wound care. Many home health agencies operate below capacity due to clinician shortages, meeting only 61% to 70% of demand and frequently declining wound care referrals. Implementing artificial intelligence–powered digital wound care solutions (DWCSs) offers an opportunity to enhance wound care programs by improving scalability and effectiveness through better monitoring and risk identification.

**Objective:**

This study assessed clinical and operational outcomes across 14 home health branches that adopted a DWCS, comparing pre- and postadoption data and outcomes with 27 control branches without the technology.

**Methods:**

This pre-post comparative study analyzed clinical outcomes, including average days to wound healing, and operational outcomes, such as skilled nursing (SN) visits per episode (VPE) and in-home visit durations, during two 7-month intervals (from November to May in 2020-2021 and 2021-2022). Data were extracted from 14,278 patients who received wound care across adoption and control branches. Projected cost savings were also calculated based on reductions in SN visits.

**Results:**

The adoption branches showed a 4.3% reduction in SN VPE and a 2.5% reduction in visit duration, saving approximately 309 staff days. In contrast, control branches experienced a 4.5% increase in SN VPE and a 2.2% rise in visit duration, adding 42 days. Healing times improved significantly in the adoption branches, with a reduction of 4.3 days on average per wound compared to 1.6 days in control branches (*P*<.001); pressure injuries, venous ulcers, and surgical wounds showed the most substantial improvements.

**Conclusions:**

Integrating digital wound management technology enhances clinical outcomes, operational efficiencies, and cost savings in home health settings. A reduction of 0.3 SN VPE could generate annual savings of up to US $958,201 across the organization. The adoption branches avoided 1187 additional visits during the study period. If control branches had implemented the DWCS and achieved similar outcomes, they would have saved 18,546 healing days. These findings emphasize the importance of incorporating DWCSs into wound care programs to address increasing demands, clinician shortages, and rising health care costs while maintaining positive clinical outcomes.

## Introduction

The demand for home health care and nursing visits has surged due to the persistent rise in the prevalence of comorbidities and the aging population [1,2]. In the United States, 2% of the population have complex chronic wounds, further driving the growing demand for home health care services [3]. Approximately, a third of patients who use home health have at least one wound [4,5], leading to the allocation of a substantial portion of the budget and resources in a home health agency (HHA) for nursing visits being dedicated to wound assessment and care [5]. Nursing visits in HHAs consume a significant proportion of health care delivery costs, primarily due to the time spent by nurses in assessing and managing wounds [6]. Studies have indicated that wound management uses, on average, about 50% to 70% of the nurses’ resources [7-11], with over 60% of their time dedicated to changing dressings [12], resulting in an average of extra 3 visits per week [7].

According to the 2019 report from the Centers for Medicare & Medicare Services (CMS), 1.6% of the US population received wound care at a Medicare-certified HHA, totaling 5,266,931 individuals with wounds [13]. This suggests that approximately 15,800,793 patient contacts for dressing changes occur per week, requiring around 7,900,396 clinician hours per week to be spent on wound care visits in HHAs [13]. Research supports the notion that effective wound care management is best achieved through a collaborative health care team [14]. However, the absence of a standardized approach to evaluating wounds and the limited communication platforms for supporting collaboration between clinicians may lead to unnecessary or prolonged visits and extended healing times [15].

Research studies have reported that wounds, especially pressure injuries, pose the highest risk factor for hospitalization, increasing the length of stay by an average of 4.31 days [16,17]. Thus, with 1 in 3 patients who use home health dealing with wounds, a focus on providing higher-quality, more efficient care for patients with wounds has the potential to lead to faster healing and reduced complications for patients, as well as a substantial cost savings and improved reimbursement for HHAs and the health care system.

The increasing number of visits, visit duration, healing time for wounds, and hospital stays have placed a significant burden on the already financially stained health care system, compounded by a shortage of specialized nurses [18]. The majority of HHAs operate below capacity due to clinician shortage, only meeting 61% to 70% of the demand for wound care, leading many to reject wound care referrals [19,20]. This crisis is partly due to inadequate allocation of resources, funding constraints at the organizational level, and the increasing number of nurses leaving the practice or retiring [18]. Thus, addressing the substantial resource demand for managing chronic wounds poses a significant challenge for these agencies [5].

In light of these challenges, it remains crucial to control costs while optimizing outcomes within the health care system. Recognizing the high complexity and resource costs of providing wound care in home health, the CMS allocated the highest base reimbursement for the wound clinical grouping under its Patient Driven Groupings Model value–based payment regime, which was rolled out nationwide in 2020 [13]. Additionally, to address the sustainable health care costs associated with unintended hospital use (such as acute care hospitalizations and emergency department visits), CMS introduced its Expanded Home Health Value-Based Purchasing Model in 2023. This program adjusts an HHA’s annual Medicare reimbursement based on the achievement of various quality measures, with the most heavily weighted measure being “unintended hospital use.”

Using a digital wound care solution (DWCS) for patients with wounds has been linked to faster healing times [21] and more efficient wound care documentation [22]. DWCSs integrate artificial intelligence (AI) to monitor wound progress and identify potential risks [23], and they are interoperable with organizational systems, allowing efficient and secure data exchange. The seamless data exchange through AI technologies [24] is crucial for establishing a cohesive wound care program that can adopt and scale up digital documentation and objective AI assessment data. Recognizing these benefits, many health care settings have transitioned to incorporating digital technologies to enhance clinician efficiency, capacity, and confidence, ultimately allowing them to deliver a higher quality of care to more who require wound care [21,22,25].

In 2021, CenterWell Home Health (CenterWell), an HHA with 355 branches across 40 states in the United States, launched a comprehensive wound care program to deliver high-quality care. The program involved providing advanced education and training for clinicians in wound management and using the DWCS Swift Skin and Wound (Swift Medical Inc) for quality wound care evaluations. The training program, known as Prevention Intervention, Management, and Education (PRIME), was designed to build the knowledge, skills, and abilities of clinicians in wound care, establishing a network of skilled wound care champions in the field. The DWCS is an evidence-aligned, AI-based technology that captures precise wound images and accurate measurements and provides predictive analytics supporting the wound escalation processes to provide ongoing performance support.

There is a lack of research evaluating the impact of integrating technology into wound care delivery within a home health setting. To our knowledge, this study represents the first attempt to outline the clinical and operational advantages of a wound care program incorporating digital technology in a home health environment. The study’s objective was to evaluate the enhancements in clinical outcomes (such as the average time required to heal a wound) and operational outcomes (including the volume of skilled nursing [SN] visits per episode [VPE] and the duration of in-home SN visits) at 14 CenterWell branches (initially scoped for this implementation and study). We examined the same 7-month period (from November to May) before (2020) and after (2021) the implementation of the DWCS as part of the comprehensive wound care model in home health. Additionally, the study compared the changes in these clinical and operational benefits with a similar control group of PRIME-certified home health branches that had not yet adopted the digital solution.

## Methods

### Study Design and Data Sources

This benefits-evaluation study used a pre-post comparative design, using wound care data captured in the Homecare Homebase (HCHB) health information system. HCHB is an electronic medical record (EMR) software developed in 1999. This software is hosted on the cloud and is designed to facilitate home health care frontline workers’ abilities to monitor their clinical outcomes and operational activities to enhance the quality of patient care.

Through the HCHB platform, a home health organization can extract a wide range of clinical and administrative data. These include admission and referral data; patient assessment details such as start date of wound care, 60-day episode start and end dates, wound types and stages; as well as the date and time of visits within the 60-day episode. Additionally, it can retrieve information about the discipline and service code of clinicians conducting the visits, discharge dates, hospitalization details, patients’ demographics, comorbidities, and payer types. The study’s focus was to extract structured data that pertain to the comprehensive management of patients with wounds. This involved filtering the data within the study periods to include records of patients with wounds in the Integumentary Command Center (ICC).

### Ethical Considerations

In this study, for the postadoption data, the DWCS used industry-standard protocols; for example, using Health Level 7 to wirelessly transfer encrypted wound care information (wound images and documentation recorded by clinicians at the participating branches using the solution) bidirectionally with HCHB in real time to ensure that outcome data could be monitored and to eliminate double documentation. Wound care data were then accessed from the EMR for tracking and analysis. All communications to the servers follow the Advanced Encryption Standards and comply with the Health Insurance Portability and Accountability Act.

Institutional review board approval was provided by CenterWell HealthCare Center for this quality improvement (QI) descriptive evaluation study. This QI study, which adheres to Tri Council Policy Statement QI policy, was granted an exemption of ethics review (ID:2023-0100) from Pearl IRB, LLC, an independent institutional review board.

### Data Abstraction Process and Sample

The anonymous wound care assessment records were collected from the ICC, an entity focused on the comprehensive management of patients with wounds, at 14 CenterWell branches where the DWCS was implemented as part of the comprehensive wound care model. Additionally, records were obtained from 27 control branches at CenterWell that had not yet adopted the DWCS into practice. The 27 control branches were carefully selected to match the criteria of the adoption branches in terms of size; geographical locations; capacity; volume of referrals; and the clinicians’ levels of clinical wound care education, training, skills, and expertise. This rigorous selection process aimed to ensure an unbiased comparison when assessing the impact of the DWCS technology on clinical and operational outcomes.

This study compared the change in clinical outcomes by analyzing the median days to heal a wound in the pre- and postadoption periods within the study timeframe for the 14 adoption sites and the 27 control sites. Additionally, operational outcomes were compared, including the number of SN VPE, the associated projected cost-savings, and the duration of in-home SN visits in the same periods for the adoption and control branches.

The study and analysis included wound assessments of all patients with any type of wound that met the following criteria:

1. There were wound records (primary and secondary diagnoses) of any type recorded on admission from both adoption and control branches at CenterWell that were referred to and managed at these sites during the 7-month study period (from November 1, 2020, to May 31, 2021, and from November 1, 2021, to May 31, 2022).
2. For the postadoption period, wounds had to be assessed and managed using the Swift Medical Inc solution at the participating 14 adoption branches in the postadoption period.
3. The records pertained to adult patients aged 18 years or older.

Any wounds outside of the study period were excluded from the analysis. Patients at adoption branches who did not receive wound care using the technology during the study period were not included in the analysis in the postadoption period. This exclusion applied to patients with closed surgical wounds, external fixators, bruising, cellulitis, and extensive diffuse dermatological conditions.

From the 14 adoption branches, we collected and included data from 5239 sixty-day wound episodes involving 3738 unique patients in 2020-2021 (preadoption period) and 3958 sixty-day wound episodes involving 2757 unique patients in 2021-2022 (postadoption period). Similarly, for the 27 control branches, the analysis incorporated data from 5592 sixty-day episodes involving 3859 unique patients in 2020-2021 (preadoption period) and 5429 sixty-day episodes involving 3924 unique patients in 2021-2022 (postadoption period).

The organization’s wound care research team (KJ, DG, and KC) independently extracted all required wound patient data for the adoption and control branches based on the wound start data and medical record number from the HCHB EMR during the first week of August 2022. Using the same instrument, filters, steps, and procedures, the eligible wound data were accessed and then deidentified for sharing with the evaluation team (HTM and DM) for analysis. Each patient was assigned a study and episode ID number, with no linkage between the medical record number and study ID developed to ensure patient anonymity.

The deidentified data, including essential wound assessment–related variables such as patients’ characteristics, episode ID, referral date, branch code, type of wound, classification and anatomical location of wound, wound care start date and effective date of care, start and end dates of episode, wound status, primary diagnosis, visit start date and time, visit end time, duration of visit, service code and description, discipline code, and payor type, were shared in a Microsoft Excel spreadsheet with the evaluation team through a secure platform.

### Statistical Analysis

We analyzed wound data in both the adoption and control groups during the pre- and postadoption periods. This analysis encompassed both numeric variables, such as patients’ age, and categorical variables, including patients’ sex, wound type, payor type, episode status, and comorbidities. In addition, the study calculated several data indictors:

- Home visit utilization, assessed based on the average number of SN visits per 60-day episode: This metric was determined by dividing the number of SN visits (numerator) by the total number of episodes cared for (denominator) at the participating branches during the study period.
- Home visit efficiency, assessed based on average time to complete an SN visit per 60-day episode: It involved calculating the mean time to complete an SN visit, measured in minutes. This calculation was based on the time lapse between the start and end time of each in-home visit per 60-day episode. This analysis considered visits conducted by skilled nurses (registered nurse [RN] and licensed practical nurse [LPN]). These visits included the following specific service description codes: *RN Oasis Admission, SN high Tech Visit-Lasting 1.5 Hours, SN Infusion Subsequent Visit, SN PRN Visit as Needed, SN Rapid Subsequent Visit,* and *SN Subsequent Visit*. As no out-of-home documentation occurs for routine and SN visits, the calculated time encompassed all patient care and documentation activities within a visit. Overall, the findings were summarized using frequencies, means, and SDs.
- Average days to heal a wound: The analysis of average days to heal a wound included any type of wound with an inactive date and considered as “healed,” as determined by CenterWell. The study collected the first and last date (inactive date) of the wound and calculated the average days (mean) to heal based on the number of days between the start date and the inactive date of healed wounds. Patients with open wounds who were discharged were not included if the wound was not known to be healed. This analysis segmented the data by wound type (ie, pressure injury, venous ulcer, etc). A Student sample two-tailed *t* test was used to examine the difference in the average number of days to heal a wound across adoption and control branches between the pre- and postadoption periods. The significance of the statistical test was accepted at*P*<.05.

Data analysis was conducted using SPSS software (version 28; IBM Corp).

The analyses showed that the days to heal for both the adoption and control groups were normally distributed, as assessed by Shapiro-Wilk normality test (*P*>.05). Additionally, there was homogeneity of variances, as assessed by the Levene test for equality of variances for both the adoption and control groups (*P*=.17 and *P*=.32, respectively).

## Results

### Overall Characteristics

The data were collected from 14,278 patients with wounds from both the adoption and control branches, all of whom were recorded in the ICC platform and fulfilled the inclusion criteria. Of these patients, 26.2% (n=3738) were from the adoption branches in the preadoption period and 19.3% (n=2757) were from the adoption branches in the postadoption period. The age of the patients ranged from 23 to 108 years, with approximately half (n=7351, 51.5%) of the participants being female. The included wounds encompassed various types, with surgical wounds and pressure injuries being the most common (Table 1). Overall, there were no statistically significant differences (*P*>.05) between the adoption and control groups in the different time periods, indicating a comparable distribution of wound types across the groups.

**Table 1:** Overall characteristics of wound records at the adoption and control branches in the pre- and postadoption periods.

Characteristics		Adoption branches		Control branchers	
		Preadoption period (November 2020 to May 2021)	Postadoption period (November 2021 to May 2022)	Preadoption period (November 2020 to May 2021)	Postadoption period (November 2021 to May 2022)
Unique patient admission, n		3738	2757	3859	3924
Age (years), mean (SD)		73.1 (13.4)	72.3 (13.7)	73.6 (13.2)	74.6 (11.6)
**Sex, n (%)^a^**					
	Male	1854 (49.6)	1331 (48.3)	1863 (48.3)	1879 (47.9)
	Female	1884 (50.4)	1426 (51.7)	1996 (51.7)	2045 (52.1)
Wounds episodes managed at participating branches, n		5239	3958	5592	5429
**Wound type, n (%)^b^**					
	Arterial ulcer	41 (0.8)	23 (0.6)	30 (0.5)	31(0.6)
	Burn	24 (0.5)	11 (0.3)	37 (0.7)	36 (0.7)
	Diabetic ulcer	254 (4.8)	120 (3.0)	326 (5.8)	285 (5.2)
	Pressure injury	1445 (27.6)	1081 (27.4)	1535 (27.4)	1483 (27.3)
	Skin tear	278 (5.3)	230 (5.8)	416 (7.4)	379 (7)
	Surgical wound	1739 (33.2)	1374 (34.7)	1406 (25.3)	1435 (26.4)
	Traumatic wound	357 (6.8)	306 (7.7)	505 (9)	489 (9)
	Venous ulcer	358 (6.8)	444 (11.2)	457 (8.2)	506 (9.3)
	Others^c^	743 (14.2)	369 (9.3)	880 (15.7)	785 (14.5)
Episodes associated with comorbidities, n (%)^b^		2664 (50.8)	2182 (55.1%)	3143 (56.2)	3202 (59)
Episodes not associated with comorbidities, n (%)^b^		2575 (49.2)	1776 (44.9)	2449 (43.8)	2224 (41)
Current episodes, n (%)^b^		396 (7.6)	270 (6.8)	178 (3.2)	225 (4.1)
Discharged episodes, n (%)^b^		2341 (44.7)	1780 (45.1)	2725 (48.7)	2615 (48.2)
Recertified episodes, n (%)^b^		2502 (47.8)	1908 (48.3)	2689 (48.1)	2589 (47.7)

^a^Percentages use the number of unique patient admissions as the denominator.

^b^Percentages use the number of wound episodes as the denominator.

^c^Other types of wounds: abrasion, laceration, blisters, seroma, carcinoma, and hematoma.

### Reduction in Utilization of SN Home Care Visits

The data show that the adoption branches experienced a decrease in the average number of SN visits per 60-day episode during the postadoption period as compared to the preadoption period. This led to a decline of 4.3% in the average number of SN VPE from the preadoption period to the postadoption period. On the other hand, the control branches saw a 4.5% increase in the average SN VPE. Consequently, the adoption branches showed an 8.7% improvement in visit utilization compared to the control branches over the same period, as illustrated in Table 2.

**Table 2:** Comparison of average skilled nursing visits per episode between the adoption and control branches in the pre- and postadoption periods.

Branches	Preadoption period (November 2020 to May 2021)			Postadoption period (November 2021 to May 2022)	
	Visits, n	Skilled nursing visits per episode, n	Visits, n		Skilled nursing visits per episode, n
Adoption	36,433	7	26,825		6.7
Control	36,969	6.6	37,678		6.9

### Improved SN Wound Care Visit Efficiency

During the preadoption period, the adoption branches spent an average of 43.2 (SD 40.27) minutes per episode, amounting to 1,573,171 minutes for completing visits. However, in the postadoption period, the average time per episode reduced to 42.1 (SD 37.52) minutes, resulting in a total time spent of 1,128,658 minutes by the adoption branches. This decrease led to a 2.5% reduction in the average time required to complete a visit. In total, the adoption group saved 309 days (equivalent to 444,513 minutes) of staff time spent on SN home visits. On the other hand, the control branches experienced a 2.2% increase in the average time to complete the SN visit, from an average of 40.9 minutes (SD 34.20) to 41.8 minutes (SD 36.37). Overall, the adoption branches saw a 4.4% improvement compared to the control group from the preadoption period to the postadoption period, as depicted in Table 3.

**Table 3:** Comparison of average time to complete a skilled nursing visit between the adoption and control branches in the pre- and postadoption periods.

Branches	Preadoption period (November 2020 to May 2021)		Postadoption period (November 2021 to May 2022)	
	Visits, n	Average time to complete a skilled nursing visit (min)	Visits, n	Average time to complete a skilled nursing visit (min)
Adoption	36,433	43.2	26,825	42.1
Control	36,969	40.9	37,678	41.8

### Improved Average Days to Heal a Wound

A significant decrease in the average healing time of wounds was observed at the adoption branches compared to the control branches (*P*<.001). On average, the adoption branches saw an average reduction of 4.3 days per wound (from 19.7 days to 15.4 days), which was greater than the control group’s average reduction of 1.6 days (from 25.9 days to 24.3 days). This corresponds to a 2.7-day improvement compared to the control group, and an overall 15.7% improvement in healing time for the adoption branches (Table 4). Additionally, significant differences were noted in the average days saved between the pre- and postadoption periods for the adoption branches, particularly the reduction in days to heal for pressure injuries, venous ulcers, and surgical wounds (*P*=.01, *P*<.001, and *P*<.001, respectively). In contrast, the average healing time for traumatic wounds, surgical wounds, and diabetic ulcers were increased from the preadoption period to the postadoption period for the control branches. No significant differences were found for any saved days for different types of wounds between the control group between the pre- and postadoption periods (all *P*>.05; Table 4).

**Table 4:** Average days to heal a wound between the adoption and control branches in the pre- and postadoption periods.

Type of wound	Adoption branches						Control branches						
	Preadoption period (November 2020 to May 2021)		Postadoption period (November 2021 to May 2022)		*P* value	Preadoption period (November 2020 to May 2021)				Postadoption period (November 2021 to May 2022)			*P* value
	Days to heal, mean (SD)	Episodes, n	Days to heal, mean (SD)	Episodes, n		Days to heal, mean (SD)		Episodes, n	Days to heal, mean (SD)		Episodes, n		
All wounds	19.7 (13.9)	2185	15.4 (15.0)	1856	*<.001^a^*	25.9 (20.4)		2770	24.3 (19.7)		2200	.98	
Diabetic ulcer	17.9 (12.6)	106	16.8 (15.4)	55	.68	34.0 (21.1)		326	34.2 (18.3)		261	.98	
Pressure injury	18.9 (13.0)	679	15.3 (14.3)	540	*.01*	30.4 (21.2)		735	29.9 (18.6)		695	.88	
Skin tear	16.8 (11.6)	72	14.3 (11.7)	80	.14	14.4 (15.2)		116	14.2 (14.6)		110	.92	
Surgical wound	20.8 (15.2)	608	15.4 (15.9)	508	*<.001*	18.3 (18.0)		514	21.3 (16.7)		345	*.01*	
Traumatic wound	20.8 (13.5)	136	17.9 (15.6)	183	.06	22.9 (19.9)		305	24.0 (19.8)		125	.19	
Venous ulcer	18.9 (13.3)	268	13.4 (15.4)	313	*<.001*	31.9 (21.1)		357	29.8 (20.5)		255	.85	
Others	21.2 (15.7)	316	17.6 (15.5)	177	.06	28.1 (20.9)		280	30.5 (19.1)		274	.32	

^a^*P*<.05.

## Discussion

### Principal Findings

To our knowledge, this descriptive, pre-post evaluation study is the first to investigate the impact of adopting digital wound management technology in a home health setting as part of a comprehensive wound care program. Overall, our study recorded a general improvement in clinical and operational benefits among the adoption branches in the postadoption period compared to the preadoption period, surpassing the control branches over the same time frame. For instance, skilled nurses in the adoption branches saved 444,513 minutes, equivalent to 309 days, spent conducting in-home wound care visits after implementing the technology in practice, while the control group added 42 days to the time spent conducting the visits. This finding is crucial for addressing the consequences of the growing shortage of trained nurses in the health care system [26] and the increasing demand to cope with the continuous rise in wound prevalence and the aging population [27].

On average, nurses provide patients with three dressing changes per week [28-30], and according to O’Keeffe [12], this takes up to 66% of the available nursing time. Also, literature has shown that up to 35% of this time is spent on documentation [26,31-34], and 21% is spent on care coordination [26,31]. In a previous study, Lindholm et al [35] stated that among a population of 694 patients with wounds, changing wound dressings alone consumed time comparable to full-time employment of about 57 nurses.

The time spent changing a dressing in a typical wound care visit is not only about the physical act of changing the dressing but also always involves other activities such as wound assessment, measurement, and decision-making on the need to change a dressing and the choice of dressing. This comprehensive approach can make the process time-consuming for nurses, as highlighted in studies by Hadcock [36] and Fletcher and Wasek [37], and could be challenging at times [37]. Moreover, the advent of electronic health records was intended to streamline wound care coordination and documentation at home health organizations [38], but studies by Burton et al [39], Sockolow et al [40], and Yang et al [38] have shown that electronic health records may only partially support these processes and can add to the nurses’ workload.

The time-saving benefits documented in our study aligns with a previous study that found implementing the Swift Medical Inc solution in an outpatient clinic could potentially save up to 51.7 days of clinicians’ time per year compared to traditional wound assessment methods [22]. The observed time saved during home visits may be attributed to the provision of a technology to nurses that helps facilitate effective wound management. The technology allowed for accurate clinical wound information and precise wound images to be captured, enabled online documentation during visits, allowed the electronic exchange of clinical information, and facilitated remote monitoring with experts. This accessibility to best practice wound assessment ultimately led to improved care and cost outcomes.

Further, after implementing the technology, the adoption branches also experienced a 4.3% reduction in the average number of SN visits needed to care for a wound per episode. This reduction in home SN VPE could generate significant cost savings in the home health care setting. Assuming the average hourly rates of LPNs and RNs conducting the SN visits in home health care range from US $26.85 to US $42.85, according to the US Bureau of Labor Statistics [41], CenterWell could potentially save US $600,413 to US $958,201 annually across the organization for every 0.3 reductions in SN VPE, based on a total of 60,898 episodes cared for at the organization in a year. Furthermore, compared to the control group, if the adoption branches had not implemented the Swift Medical Inc solution, they would have conducted an additional 1187 visits during the study period.

Evidence suggests that incorporating technology into wound care management can lead to substantial cost savings by reducing nurses’ transportation costs and the utilization of wound care materials with each additional visit [42]. Additionally, Lindholm and Searle [5] demonstrated in a cost-effectiveness study that saving 260 hours of nurses’ time per year could result in up to an 80% reduction in management costs. This, in turn, could lead to a reduction in care delivery costs and an increase in practice capacity, allowing for better resource management and reduced workload on clinicians, which may mitigate costs associated with staff burnout, attrition, and recruitment [43]. The time saved could be redirected to managing other patients or engaging in valuable activities [5]. A survey study indicated that clinicians expressed a strong interest in using any saved time to educate current patients on dressing change techniques, thus creating an opportunity for additional time savings that could be allocated to care coordination [37].

It is important to note that despite conducting fewer visits per wound episode and saving time during each visit at the adoption branches, the quality of care provided at these branches remained consistently high. In fact, there was a significant improvement in the average days to heal a wound between the pre- and postadoption periods at the adoption branches. Several research studies have shown that chronic wounds often have extended healing times, which can lead to increased consultation time, treatment supply consumption, dressing changes, and assessment sessions [44-48]. Moreover, prolonged healing times can increase the risk of complications and hospitalization, ultimately adding to the total cost of wound care [44-48]. As a result, time to heal could be the principal driver in reducing total wound care costs [45-48]. Our findings revealed a 15.7% reduction in the average days to heal a wound for the branches that adopted the technology from the preadoption period to the postadoption period (*P*<.001), resulting in a savings of 4.3 days for the adoption branches and only 1.6 days for the control branches. It is projected that if the control branches had adopted the solution and experienced the same improvement as the adoption branches, they would have saved 18,546 days in healing patients with wounds over the same period.

The management of wound care involves a variety of practices including assessment, treatment delivery, utilization of advanced products, services, and supportive tools to improve skills and optimize wound care and management [49-51]. The inclusion of AI technology in wound care can significantly enhance clinicians’ ability to manage patient care through precision, efficiency, and interoperability. A study by Chairat et al [24] demonstrated that AI integration can streamline documentation and ensure smooth data exchange across health care systems. Research also has demonstrated that AI algorithms, when applied to wound images captured by smart devices, can achieve over 90% accuracy in identifying wound types and dimensions, streamlining the documentation process and ensuring seamless data exchange across health care systems [24]. Therefore, integrating technology as an essential component of the program is crucial for enhancing wound care, as evidenced by various studies [44,49-52].

Our research indicates that implementing wound care management technology enhances patient outcomes and contributes to cost containment and savings. This aligns with the estimated US $15.7 trillion expansion of the global economy by 2030 attributed to the implementation of AI-based technologies, including assisted intelligence, automation, and autonomous intelligence [53]. Additionally, staying current with wound care knowledge and advancements and integrating technology can significantly improve proficiency in wound care and assist evidence-informed clinicians in delivering effective treatment recommendations, irrespective of wound complexity or clinician expertise.

Chronic wounds are often tied to comorbid conditions, increasing the complexity of wound management and placing more burdens on clinicians and patients [46]. Meanwhile, CMS expects HHAs to take on more responsibility in caring for these patients with clinically complex conditions and bridge the referral gap in this population [13].

Hence, it is essential to modernize health care and implement wound management technology to bolster evidence-based practices and enhance clinical management [50], regardless of the wound complexity or the practitioners’ experience [50,54-56]. This can be accomplished through the practical implementation of deep learning for automated tracing of wound dimensions, accurate measurements [22], automated wound tissue segmentation [57], and predictive modelling [23], thus enabling real-time decision-making that ensures timely patient-centered care.

As evidenced by our study, the branches that adopted the digital solution demonstrated improvements in managing more complex wounds with comorbid conditions, showing a 1.5% increase compared to the control branches. Additionally, these branches reported a 5% improvement in the rate of healed wounds from the preadoption period to the postadoption period, contrasting with the observed 9% decrease in the overall rate of healed wounds at the control branches. These findings illustrate a modest improvement in the effectiveness of using advanced wound management solutions that have a potentially larger impact if adopted across additional branches within the enterprise. Therefore, integrating AI-powered wound management technology into a comprehensive wound care model enables better wound care delivery and management.

### Limitations and Strengths

A major strength of this study is the inclusion of patients who require wound care managed at 41 different branches at one of the largest HHAs in the United States. We also included a control group of 27 PRIME-certified branches, allowing us to compare operational and clinical changes and marginal benefits against them for the same periods. However, while the branches (adoption and control groups) were comparable with regard to demographics, clinical variables, and wound care education and training, generalizing the results warrants caution. Other institutions may not be similar in size, patient demographics, and operational and clinical workflow, so the results should be interpreted within this context.

We used the as-treated analysis approach to gain valuable insight into the impact of implementing the Swift Medical Inc solution in practice. To ensure the accuracy of our findings, we excluded patients who had not undergone assessment using the Swift Medical Inc solution at the adoption sites during the study period. This approach provided a focused understanding of the true impact of the technology on the assessed outcomes.

It is important to note that this study was conducted within a specific time frame of 7 months (from November to May) in both 2020-2021 and 2021-2022. The COVID-19 pandemic significantly disrupted priorities and various aspects of the wound care continuum in 2020, as highlighted by Sen [58]. The increased infection rates of the respiratory pathogen among populations with comorbidities prompted heightened attention to high-risk patients [58] in the preadoption period, and this may have had an unforeseen impact on our findings, either positive or negative.

In addition, due to a lack of data, the study did not assess the sociodemographic variables of patients with wounds, diagnostic methods, nurses’ travel costs, cost of used wound supplies, patient costs, or quality of life. Including these variables in exploring the technology’s cost-effectiveness would provide valuable insight into the potential savings associated with technology in wound care. A future comprehensive cost-effectiveness study that includes all these variables would be beneficial in informing policy makers and payers about the tangible economic impact of adopting technology for home health to reduce care costs and improve patient outcomes.

Nevertheless, this study presented preliminary data on the impact of adopting a comprehensive wound care management model with the inclusion of technology. Our findings illustrated that HHAs could realize cost savings and clinical and operational improvements by integrating this technology into the wound care program. Therefore, the results may hold significant value for health care providers, administrators, policy makers, and insurance companies.

### Conclusion

Incorporating wound management technology into the wound care paradigm can improve operational efficiencies in home health settings by reducing the time required to complete in-home visits and decreasing the volume of SN VPE. These benefits can lead to significant cost savings. In addition, this approach also supports more effective clinical care, leading to faster wound healing, which facilitates the managing of more wound episodes annually, ultimately increasing revenue.

There is a clear need to establish a standardized comprehensive approach that incorporates digital tools as part of the wound care program. Doing so can help address challenges related to wound care assessment, increasing demands, limited human health resources, and increased burnout within the health care setting.

Furthermore, by following Centerwell’s example of enhancing clinicians’ wound care knowledge and skills with the aid of wound care management technology, other home health care organizations can achieve similar results. This includes reducing the average healing time of wounds by 27% and saving clinicians approximately 530 days annually that would have been spent on conducting more in-home wound care visits.

## Abbreviations

AI: artificial intelligence
CenterWell: CenterWell Home Health
CMS: Centers for Medicare & Medicare Services
DWCS: digital wound care solution
EMR: electronic medical record
HCHB: Homecare Homebase
HHA: home health agency
ICC: Integumentary Command Center
LPN: licensed practical nurse
PRIME: Prevention Intervention, Management, and Education
QI: quality improvement
RN: registered nurse
SN: skilled nursing
VPE: visits per episode
